# An Investigation Into the Acceptability of the SAFER-YMH Care Bundle for Transitions Out of CAMHS Crisis and Liaison Services

**DOI:** 10.1192/bjo.2024.164

**Published:** 2024-08-01

**Authors:** Josephine Holland, Pallab Majumder, James Roe, Neve Jones, Kapil Sayal

**Affiliations:** 1 University of Nottingham, Nottingham, United Kingdom; 2 Nottinghamshire Healthcare NHS Foundation Trust, Nottingham, United Kingdom

## Abstract

**Aims:**

The NHS long-term plan focuses on the improvement of Child and Adolescent Mental Health (CAMHS) community services including the roll out of 24/7 Crisis teams universally across the country. Crisis and Liaison teams form an important alternative to inpatient admission, offering intense, short-term support to young people in mental health crisis and often high levels of risk. The number of referrals to Crisis and Liaison services are rising. In order to maintain patient flow and meet demand, these teams also need safe, evidence-based protocols for efficient discharge, transition and handover of young people to community teams and services. The SAFER care bundle was designed to facilitate discharges from hospital, and this has been adapted to the SAFER-YMH bundle for discharges from adolescent mental health wards. A similar care bundle for discharge from teams offering alternatives to inpatient care has not yet been developed.

This study aimed to investigate the acceptability and necessary adaptations required for the use of the SAFER-YMH care bundle to facilitate transitions out of CAMHS Crisis and Liaison teams.

**Methods:**

This study used stakeholder feedback from multiple sources through focus groups to adapt the SAFER-YMH care bundle for use in young people in transitions out of CAMHS Crisis and Liaison teams. Normalisation process theory was utilised as the theoretical foundation upon which the development of the adapted care bundle, and its potential implementation in the complex multifaceted healthcare landscape was based.

**Results:**

Initial focus groups were held with young people, parents/carers, healthcare professionals from CAMHS crisis and liaison teams, CAMHS NHS management, NHS IT services, community CAMHS teams and NHS commissioners in two trusts in England. Following each focus group adaptations were made to the care bundle in an iterative manner. In the second round of focus groups, the adapted care bundle was presented to a mixed group of participants and agreed to be acceptable.

**Conclusion:**

Through stakeholder feedback this study has adapted the SAFER-YMH to create the SAFER-YCL care bundle; an acceptable version for use in discharges from CAMHS crisis and liaison services. End-user design and involvement is vital in the development of clinical applications and pathways which are user-friendly and time-saving for healthcare professionals and also helpful for young people and their families.

